# Differences in the position of endometriosis-associated and non-associated ovarian cancer relative to the uterus

**DOI:** 10.1186/s13244-023-01468-9

**Published:** 2023-08-15

**Authors:** Tsukasa Saida, Kensaku Mori, Toshitaka Ishiguro, Yukihisa Saida, Toyomi Satoh, Takahito Nakajima

**Affiliations:** 1 Department of Radiology, Faculty of Medicine, University of Tsukuba, 1-1-1 Tennodai, Tsukuba, Ibaraki 305-8575 Japan; 2 Department of Radiology, St. Luke’s International Hospital, 9-1 Akashi-cho, Chuo-ku, Tokyo 104-8560 Japan; 3 Department of Obstetrics and Gynecology, Faculty of Medicine, University of Tsukuba, 1-1-1 Tennodai, Tsukuba, Ibaraki 305-8575 Japan

**Keywords:** Ovary, Carcinoma, Magnetic resonance imaging, Location

## Abstract

**Background:**

Preoperative assessment of the histological type of ovarian cancer is essential to determine the appropriate treatment strategy. Tumor location may be helpful in this regard. The purpose of this study was to compare the position of endometriosis-associated (EAOCs) and non-associated (non-EAOCs) ovarian cancer relative to the uterus using MRI.

**Methods:**

This retrospective study included patients with pathologically confirmed malignant epithelial ovarian tumors who underwent MRI at our hospital between January 2015 and January 2023. T2-weighted images of the sagittal and axial sections of the long axis of the uterine body were used for the analysis. Three blinded experienced radiologists independently interpreted the images and assessed whether the ovarian tumor was attached to the uterus, and the angle between the uterus and the tumor was measured. The presence of attachment and the measured angles were compared for each histology. In addition, the angles between EAOCs, including endometrioid carcinomas (ECs) and clear cell carcinomas (CCCs), were compared with non-EAOCs.

**Results:**

In total, 184 women (mean age, 56 years; age range, 20–91 years) were evaluated. High-grade serous carcinomas (HGSCs) were significantly smaller than the others and had significantly less uterine attachment than CCCs (*p* < 0.01 for all readers). According to the mean of the measured angles, CCCs were positioned significantly more posteriorly than HGSCs and mucinous carcinomas (*p* < 0.02), and EAOCs were positioned significantly more posteriorly to the uterus than non-EAOCs (*p* < 0.01).

**Conclusion:**

HGSCs are often not attached to the uterus, and EAOCs are positioned more posteriorly to the uterus than non-EAOCs.

**Critical relevance statement:**

High-grade serous carcinomas were often not attached to the uterus, and endometriosis-associated ovarian cancers were positioned more posteriorly to the uterus than non-endometriosis-associated ovarian cancers.

**Key points:**

• The position of the ovarian tumor can be determined using MRI.

• High-grade serous carcinomas had less attachment to the uterus.

• Endometriosis-associated cancers were positioned more posteriorly to the uterus.

• The location of ovarian tumors is helpful in estimating histology.

**Graphical abstract:**

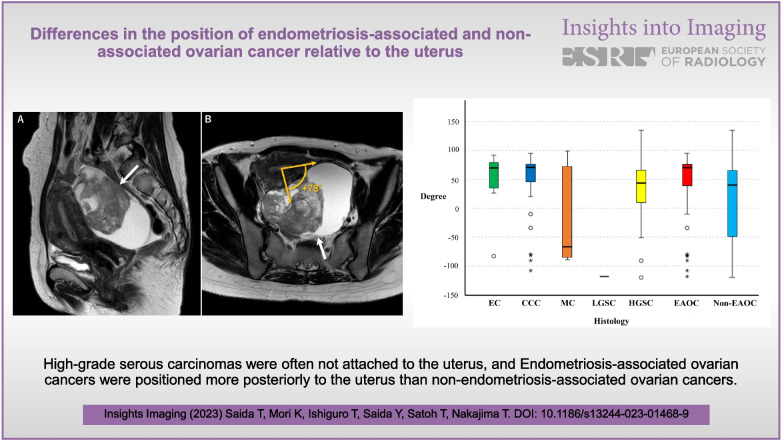

**Supplementary Information:**

The online version contains supplementary material available at 10.1186/s13244-023-01468-9.

## Introduction

Ovarian cancer is the most common fatal gynecologic malignancy and the fifth most frequent cause of cancer-related mortality in women in the United States. In 2019, approximately 13,980 deaths were expected [1]. MRI is the best radiologic method for differentiating malignant and benign ovarian tumors, and recently the American College of Radiology Ovarian-Adnexal Reporting and Data System (O-RADS) MRI committee published a lexicon and risk stratification system for adnexal lesions [2]. O–RADS MRI scores range from 0 to 5, with 0 indicating an incomplete evaluation; 1 indicating a normal ovary; 2 indicating a pure cystic mass, pure fatty mass, or pure endometriotic cyst that is almost certainly benign; 3 indicating a low risk: a cystic tumor with no enhancing solid tissue or a tumor containing solid tissue with a low-risk time–intensity curve; 4 indicating intermediate risk: a tumor containing solid tissue with an intermediate-risk time–intensity curve; and 5 indicating high risk: a tumor containing solid tissue with a high-risk time–intensity curve. O-RAD MRI has been reported to have high accuracy, sensitivity, specificity, and negative predictive value for indicating the risk of malignancy [3].

Ovarian carcinogenesis models based on histopathological, molecular biological, and genetic studies have divided ovarian carcinomas into two broad categories: type I, where precursor lesions in the ovary have been described, including low-grade serous carcinoma (LGSC), mucinous carcinoma (MC), endometrioid carcinoma (EC), clear cell carcinoma (CCC), seromucinous carcinoma, and malignant Brenner tumor; type II, where tumors may develop de novo from the tubal and/or ovarian surface epithelium, comprising high-grade serous carcinoma (HGSC), undifferentiated carcinoma, and carcinosarcoma [4]. Type II tumors have marked chromosomal instability, whereas type I tumors are genetically stable. However, they are not uniform within the same type. For example, unlike other type I tumors, CCCs are not graded and are generally considered high-grade [4]. Because of these different tumor characteristics for each histological type, it is necessary not only to differentiate between benign and malignant tumors, but also to estimate the histology.

The following are the imaging characteristics of the various histological types of ovarian cancer reported to date: HGSCs typically tend to appear as small, bilateral, predominantly solid masses with higher signal intensity on diffusion-weighted imaging and are often accompanied by peritoneal dissemination; MCs tend to be multicystic and can be very large; and the signal intensity of the mucinous content is variable and has a stained glass-like appearance. ECs and CCCs are endometriosis-associated ovarian cancers (EAOCs); therefore, coexisting endometriosis may be the key finding for these subtypes [5]. We speculated that relatively small HGSCs often do not attach to the uterus, while EAOCs are located in the cul-de-sac and adhere to the posterior uterine surface. This phenomenon would lead to differences in tumor location for each histological type; however, to date, there have been no reports examining this occurrence, and it may provide a convenient differentiation aid for radiologists who are unfamiliar or inexperienced with gynecologic MRI diagnosis. This study aimed to compare the position of each histologic type of ovarian cancer relative to the uterus using MRI.

## Materials and methods

### Patients

**Fig. 1:**
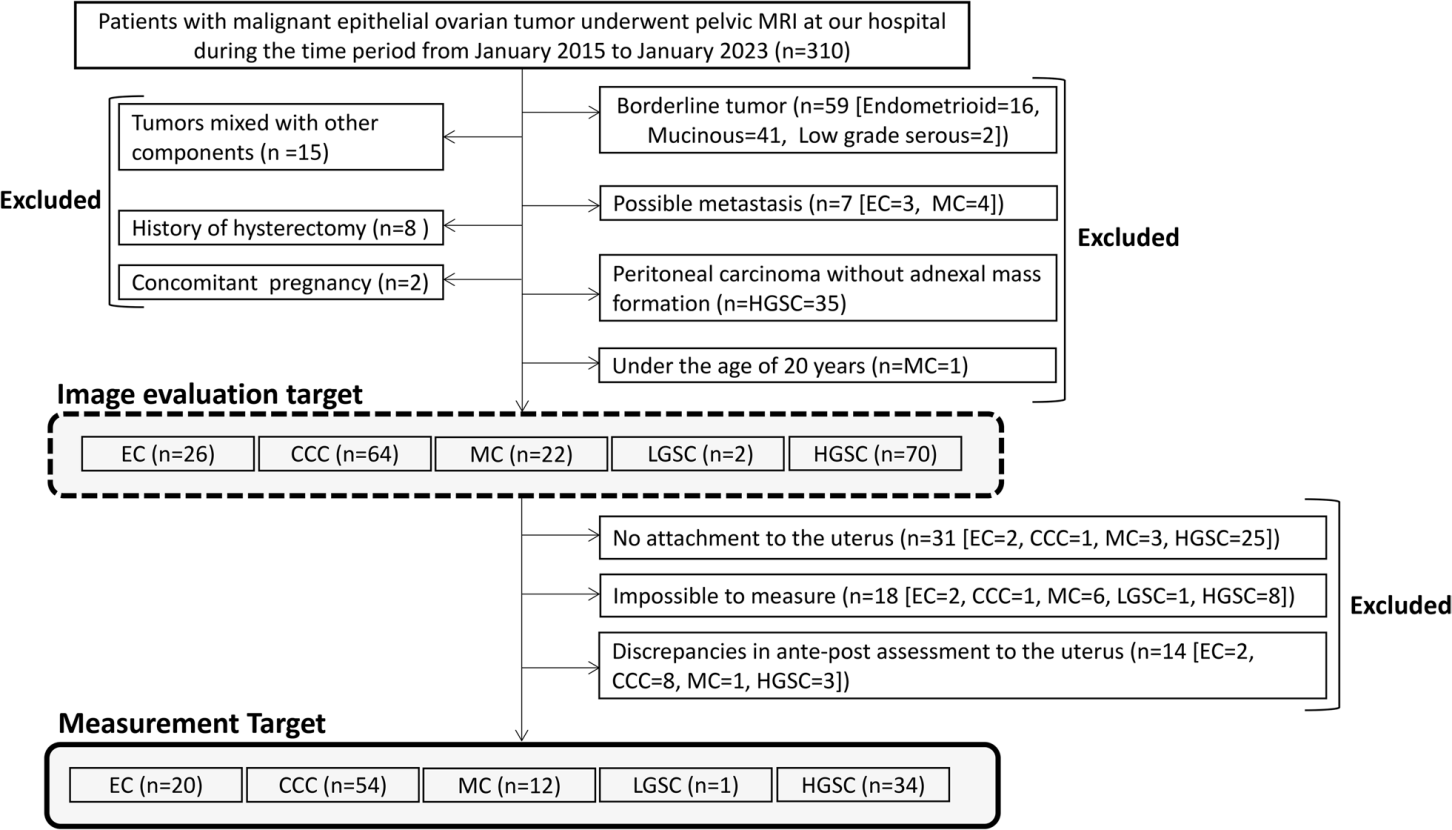
Flowchart for the patient selection process. CCC, clear cell carcinoma; EC, endometrioid carcinoma; HGSC, high-grade serous carcinoma; LGSC, low-grade serous carcinoma; MC, mucinous carcinoma

This retrospective study was approved by the Institutional Review Board of University of Tsukuba Hospital, and the need for written informed consent was waived (approval number: R04-204). The inclusion criteria were as follows: (a) women aged > 20 years for ethical reasons; (b) patients who underwent MRI, including T2-weighted images in the sagittal and oblique axial direction, performed at our hospital between January 2015 and January 2023; and (c) patients who had undergone radical surgery and had pathologically confirmed malignant epithelial tumors. To simplify the examination of histological differences in ovarian cancer, we excluded the following cases: (a) borderline tumors, (b) tumors mixed with other components such as mixed carcinomas, and (c) cases with residual possibilities of metastasis as determined by pathological examination (e.g., simultaneous cancers of the uterus and ovaries where the primary site cannot be identified). Furthermore, to investigate the relationship with the uterus, we excluded (d) cases with a history of hysterectomy, (e) cases with concurrent pregnancy, and (f) cases of peritoneal carcinoma (due to the absence of adnexal tumor formation). All eligible patients who did not meet any of the exclusion criteria and fulfilled the inclusion criteria were included in the study. A flowchart of the patient selection process is shown in Fig. 1.

MRI was performed using 3 T or 1.5 T equipment (Ingenia^®^, Achieva^®^; Philips Medical Systems, Netherlands). The images to be evaluated were 2D-T2-weighted images of the sagittal and oblique axial directions perpendicular to the long axis of the uterine body. T2-weighted images were obtained using a fast spin-echo method with the following parameters: repetition time, 1440–3571 ms; echo time, 90–110 ms; flip angle, 90°; section thickness, 3–8 mm; intersection gap, 0.3–2.0 mm; field of view, 26–48 cm; and matrix, 512 × 512 to 672 × 672.

### Clinical and pathological findings

One lesion per case was targeted; in bilateral cases, the side with the larger lesion was targeted. Based on the pathology report, the right and left sides of the ovarian tumor were identified, and the long diameter of the tumor was measured by one radiologist (14 years of experience) using MRI. The pathological presence of endometriosis was extracted from the medical records. The presence of endometriosis is defined as one of the following three conditions: i) detection of endometriosis and ovarian cancer in the same ovary; ii) detection of endometriosis in one ovary and ovarian cancer in the other; and iii) coinciding identification of ovarian cancer in any of the ovaries and pelvic endometriosis [6]. Age, menopausal status, parity, and history of abdominal surgery, including cesarean section, were also extracted from the medical records.

### Radiologist interpretation

**Fig. 2:**
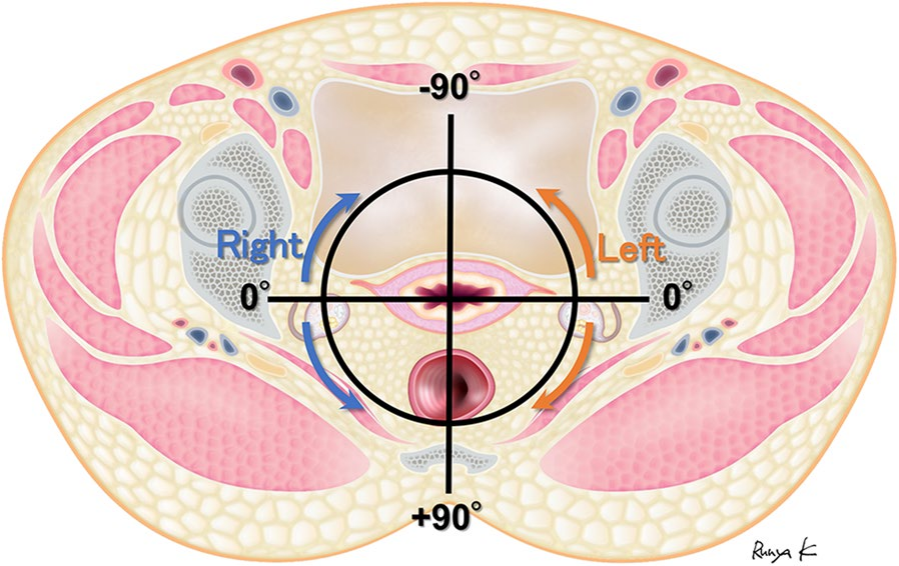
Schema for measuring angles of the uterus and ovarian tumors

Three radiologists subspecializing in abdominal MRI with 24, 14, and 12 years of post-Broad certification experience independently reviewed the T2-weighted images for each case in Centricity Universal Viewer (GE Healthcare, Chicago, Illinois, United States). All three interpreters were blinded to the pathological and clinical findings. First, the presence of an attachment between the tumor and the uterus was evaluated. If absent, accurate angle measurements could not be obtained; therefore, they were excluded from the measurement target. If present, the interpreters reviewed the axial section to the long axis of the uterine body and measured the angle between the coronal line of the uterus and the line connecting the center of the endometrium and the point of contact with the tumor. The measured angle was expressed as −180° to + 180°, with the anterior to the uterus as − and the posterior to the uterus as + . A schematic representation of the measurement of the angle is shown in Fig. 2. If an attachment was detected, but the endometrium and tumor were not in the same cross section, the measurement was judged impossible. Additionally, if it was difficult to determine the ante-post assessment and there was no agreement among the interpreters, the patients were excluded from the measurement target due to the potential for large differences in angle measurements. Furthermore, the patients were excluded from the measurement target if one of the interpreters judged a case as having no attachment to the uterus or if it was deemed impossible to measure. Moreover, the tilt of the uterus, whether anteverted or retroverted, was determined by the interpreters and decided by majority vote. A retroverted uterus was defined as a state in which the uterine body was tilted backward with respect to the cervix, while an anteverted uterus was defined as a state in which the uterine body was tilted forward with respect to the cervix or had no tilt.

### Statistical analysis

In the evaluation target, for age and tumor size, mean and standard deviations (SDs) were calculated, and comparisons between histologies were made using the Kruskal–Wallis test with the Dunn–Bonferroni post hoc test. The Kruskal–Wallis test is a non-parametric test used to compare quantitative data of three or more groups. The study also used the Dunn–Bonferroni post hoc test to determine which groups were significantly different from each other. In addition, the association of histology with the presence of the tumor and uterine attachments, impossible to measure, and discrepancy between ante-post assessment were compared using the Fisher–Freeman–Halton exact test with the Bonferroni correction. The Fisher–Freeman–Halton exact test is a non-parametric test used for qualitative data with three or more groups. The Bonferroni correction is a method used to adjust the significance level of multiple comparisons to reduce the probability of making a type I error. For the ante-post assessment, inter-reader agreement was also assessed using kappa (*κ*) statistics. The k-statistic interpreted the agreement as follows: less than 0, no; 0–0.20, slight; 0.21–0.40, fair; 0.41–0.60, moderate; 0.61–0.80, substantial; 0.81–1.00, almost perfect. In the measurement target, the mean of the angles measured by the three interpreters was used for analysis, and mean and SDs for the angles were calculated, and compared in relation to histology using the Kruskal–Wallis test with the Dunn–Bonferroni post hoc test, as well as for age and tumor size. The angles between EAOCs, including ECs and CCCs, and non-EAOCs, including MCs, LGSCs, and HGSCs, were compared using the Mann–Whitney U test, which is a non-parametric test used for quantitative data with two groups. The presence of endometriosis, uterine tilt, menopausal status, parity, history of cesarean section, and other abdominal surgery were compared to each histology and the measured angles using the Fisher–Freeman–Halton exact test with Bonferroni correction or the Kruskal–Wallis test with the Dunn–Bonferroni post hoc test. In addition, correlation coefficients were calculated between the angles or their absolute values and the overall tumor size or each histology using the Pearson correlation coefficient.

All statistical analyses were performed using the SPSS software (SPSS Statistics 28.0; IBM, New York, NY, USA). Statistical significance was set at *p* < 0.05.

## Results

A total of 184 women (mean age, 56 years; age range, 20–91 years) were evaluated.

Table 1 shows the patient and tumor characteristics for each histological type of the evaluation target. No significant differences in age were observed for any histological type (*p* = 0.08). On the other hand, there was a significant difference in size, and MCs were significantly larger than ECs, CCCs, and HGSCs (*p* < 0.01), and HGSCs were significantly smaller than CCCs (< 0.01) according to Dunn–Bonferroni post hoc test. The number of cases judged by the three interpreters as having no uterine attachment was 4–8% for EC, 2% for CCC, 5–14% for MC, 0% for LGSC, and 24–34% for HGSC, with HGSC being the most common, and a significant difference between HGSCs and CCCs was observed by all interpreters (*p* < 0.01 for all readers) after Bonferroni correction. No significant differences were found among the other histological types. One case of CCC, which was judged by all interpreters to have no uterine attachment, was unrelated to endometriotic cyst, and instead, was of adenofibroma origin. On the other hand, two of EC were judged by one or more interpreters as having no uterine attachment, and both had endometriosis. The Bonferroni correction revealed that impossible to measure cases occurred significantly more often in LGSCs than in CCCs and ECs according to reader 2 and significantly more often in MCs and LGSCs than in CCCs according to reader 3, however, the number of LGSC was small and the statistical power was insufficient. Many of the cases that were impossible to measure were those in which the tumor was attached to the uterus; however, its center was far from the uterus, thus it was not shown on the same axial image. The agreement between readers' ante-post assessment was 0.79 between readers 1 and 2, 0.79 between readers 1 and 3, and 0.77 between readers 2 and 3, indicating a substantial agreement. Most cases with discrepancies in ante-post assessment were mainly those in which the tumor was attached just above the uterus, making it difficult to perform ante-post assessment, and some cases were difficult to determine due to uterine deformation caused by myoma. No significant differences were found in this regard according to the histological type. Consequently, 63 evaluation target cases were excluded from the measurement target.

**Table 1:** Characteristics of patients and tumors of the evaluation target

Variables	EC	CCC	MC	LGSC	HGSC	All	*P* value for histology
Patients (n)	26	64	22	2	70	184	
Mean age ± SD (y)	61 ± 13	53 ± 12	53 ± 22	51 ± 17	59 ± 12	56 ± 14	0.08
Age range (y)	35–79	29–81	20–91	34–68	28–87	20–91	
Tumor size ± SD (cm)	11 ± 5	14 ± 8	21 ± 8	14 ± 3	9 ± 5	12 ± 8	< 0.01*
No contact to the uterus (n); reader1/2/3	2/1/1	1/1/1	2/1/3	0/0/0	24/17/17	31	< 0.01*/ < 0.01*/ < 0.01*
Impossible to measure (n); reader1/2/3	1/0/3	0/0/1	2/2/4	0/1/1	3/6/8	18^†^	0.11/ < 0.01*/ < 0.01*
Ante/post-discrepancy (n)	2	8	1	0	3	14	0.98

*CCC*, clear cell carcinoma; *EC*, endometrioid carcinoma; *HGSC*, high-grade serous carcinoma; *LGSC*, low-grade serous carcinoma; *MC*, mucinous carcinoma; *SD*, standard deviation

*p < 0.05

^†^Number of duplicate cases included in the no uterine contact subtracted from the impossible to measure

**Fig. 3:**
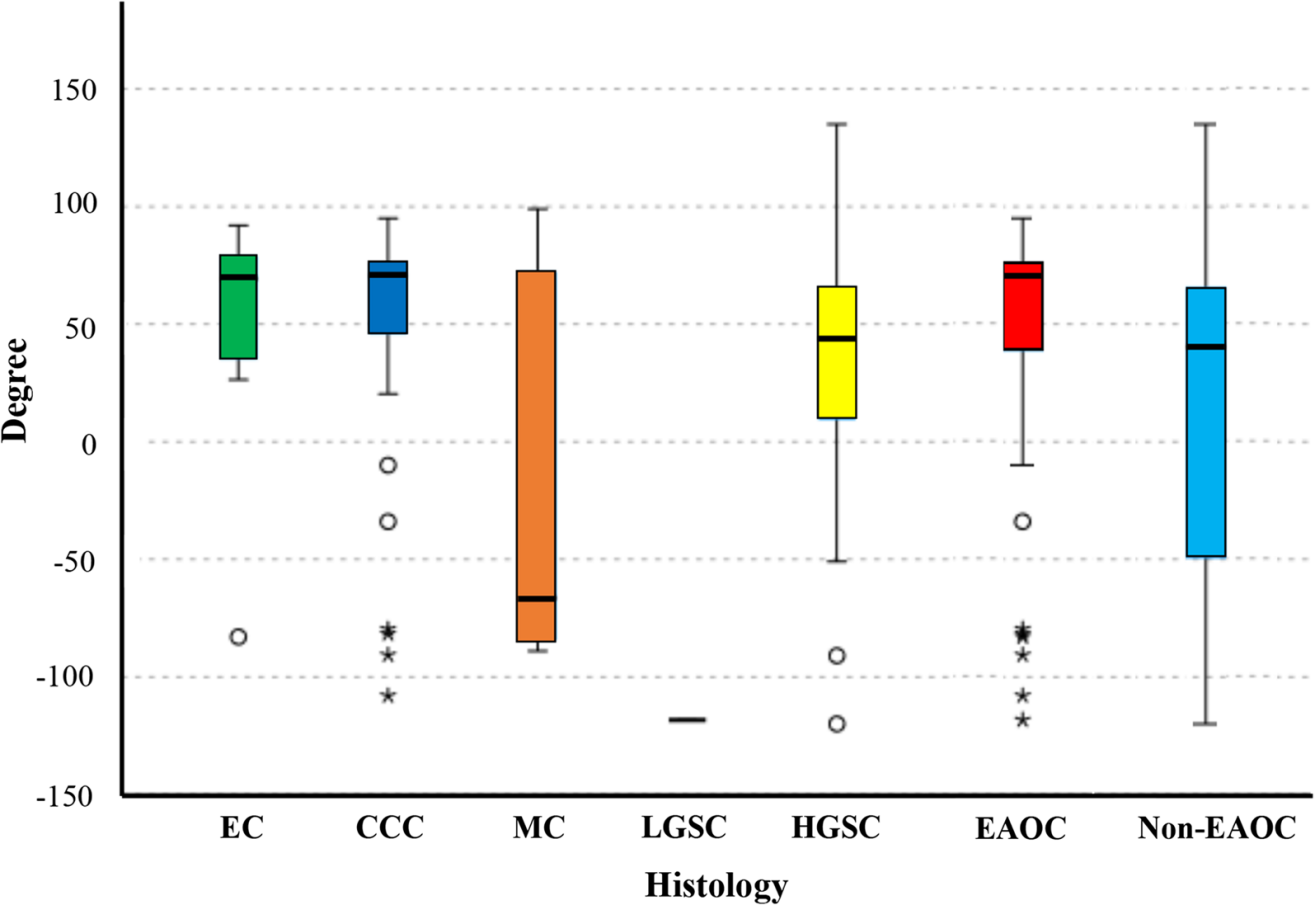
The mean of the interpreters' measured angles in a box-and-whisker diagram. MC, HGSC, and non-EAOC, including MC, LGSC, and HGSC, are widely distributed and have lower angles than EC, CCC, and EAOC, including both EC and CCC, which are relative located just behind the uterus. CCC, clear cell carcinoma; EAOC, endometriosis-associated ovarian cancer; EC, endometrioid carcinoma; HGSC, high-grade serous carcinoma; LGSC, low-grade serous carcinoma; MC, mucinous carcinoma

Table 2 shows the patient and tumor characteristics and the measured angles of the measurement target. A total of 121 women (mean age, 54 years; age range, 20–82 years) were included. Patients with EC were significantly older than the patients with CCC and MC (*p* = 0.02 and 0.02, respectively) according to the Dunn–Bonferroni post hoc test. Similar to the evaluation target, there was a significant difference in size, and MCs were significantly larger than ECs, CCCs, and HGSCs (*p* < 0.01), and HGSCs were significantly smaller than CCCs (*p* < 0.01) according to Dunn–Bonferroni post hoc test. Regarding the measured angles, CCCs were significantly positioned more posteriorly to the uterus than HGSCs and MCs (*p* = 0.01 and 0.02, respectively), and however, no significant difference was found between ECs and MCs (*p* = 0.05) or ECs and HGSCs (*p* = 0.08) (Additional file 1: Table S1). For EAOCs versus non-EAOCs, EAOCs were positioned more posteriorly to the uterus than non-EAOCs. Endometriosis was significantly more common in ECs and CCCs than in MCs and HGSCs (*p* < 0.01); however, there was no significant difference between the angles and the presence of endometriosis (*p* = 0.06). An anteverted uterus was found to be significantly more common in CCCs than in MCs (*p* < 0.01). However, no significant differences in uterine tilt were observed among the other histologic types. A significantly larger measurement angle was observed with an anteverted uterus. CCCs had a significantly lower parity than HGSCs (*p* < 0.01), but there was no significant difference in parity and the measured angles (*p* = 0.46). There was no significant association between menopausal status and experience with cesarean section or abdominal surgery and histologies or the angles. Figure 3 shows a box-and-whisker diagram of the measured angles for each histology, showing that CCC, EC, and EAOC are located relatively close to + 90° (perpendicular posterior to the uterus), while MC, HGSC, and non-EAOC are widely distributed and have a lower angle than CCC, EC, and EAOC. As for the correlation coefficients between the measured angles and their absolute values and the size of the overall tumors and each histological type, no correlation was found for any of the measured angles; however, for the absolute values, a moderate positive correlation was observed for the overall tumors (*r* = 0.444, [*p* < 0.01]), strong for HGSC (*r* = 0.582, [*p* < 0.01]), and weak for CCC (*r* = 0.284, [*p* = 0.04]).

**Table 2:** Patient/tumor characteristics and the measured angles of the measurement target

Variables	EAOC		Non-EAOC			All	*P* value for histology	*P* value for the angles
	EC	CCC	MC	LGSC	HGSC			
Patients (n)	20	52	12	1	34	121		
Mean age ± SD (y)	61 ± 14	53 ± 13	48 ± 20	34 ± 0	53 ± 10	54 ± 14	0.05*	
Tumor size ± SD (cm)	10 ± 4	13 ± 5	23 ± 8	16 ± 0	10 ± 5	13 ± 7	< 0.01*	
Angles ± SD for each histology (°)	55 ± 38	52 ± 47	−15 ± 78	−118 ± 0	31 ± 54	38 ± 57	0.01*	
Angles ± SD for EAOC or non-EAOC (°)	52 ± 45	16 ± 66	38 ± 57	< 0.01*				
Presence of endometriosis (n)	13 (65)	41 (76)	1 (8)	1 (100)	3 (9)	59 (49)	< 0.01*	0.06
Postmenopausal (n)	16 (80)	27 (53)	5 (42)	0	18 (53)	66 (55)	0.08	0.09
Anteverted uterus (n)	16 (80)	46 (88)	5 (42)	1 (100)	22 (65)	90 (74)	< 0.01*	< 0.01*
Multiparous (n)	14 (70)	28 (54)	7 (58)	0	29 (85)	78 (64)	< 0.01*	0.46
History of cesarean section (n)	3 (2)	4 (8)	2 (17)	0	2 (6)	11 (9)	0.48	0.38
History of abdominal surgery (n)	2 (1)	7 (13)	2 (17)	0	4 (12)	15 (12)	0.94	0.29

Parentheses indicate percentages

*CCC*, clear cell carcinoma; *EC*, endometrioid carcinoma; *HGSC*, high-grade serous carcinoma; *LGSC*, low-grade serous carcinoma; *MC*, mucinous carcinoma; *SD*, standard deviation

*p < 0.05

**Fig. 4:**
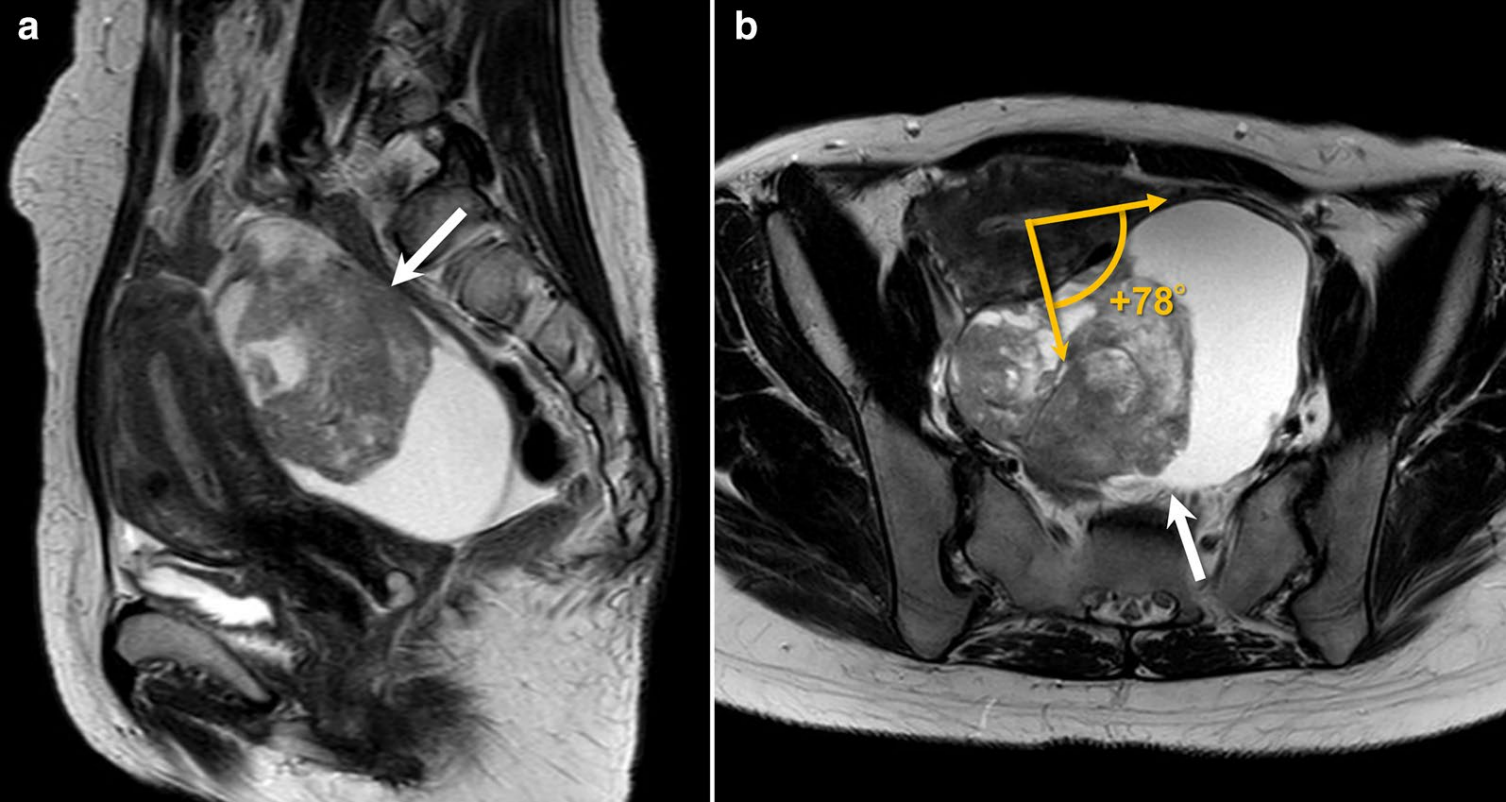
A 44-year-old woman with clear cell carcinoma. **a** Sagittal plane and **b** axial plane to the uterus show a unilocular cystic mass containing a massive solid component (arrows) widely adherent to the posterior surface of the slightly retroverted uterus. Pathologically, the mass was diagnosed as originating from the left ovary, and endometriosis was identified. The angle with the uterus is measured as + 78, as shown in B

**Fig. 5:**
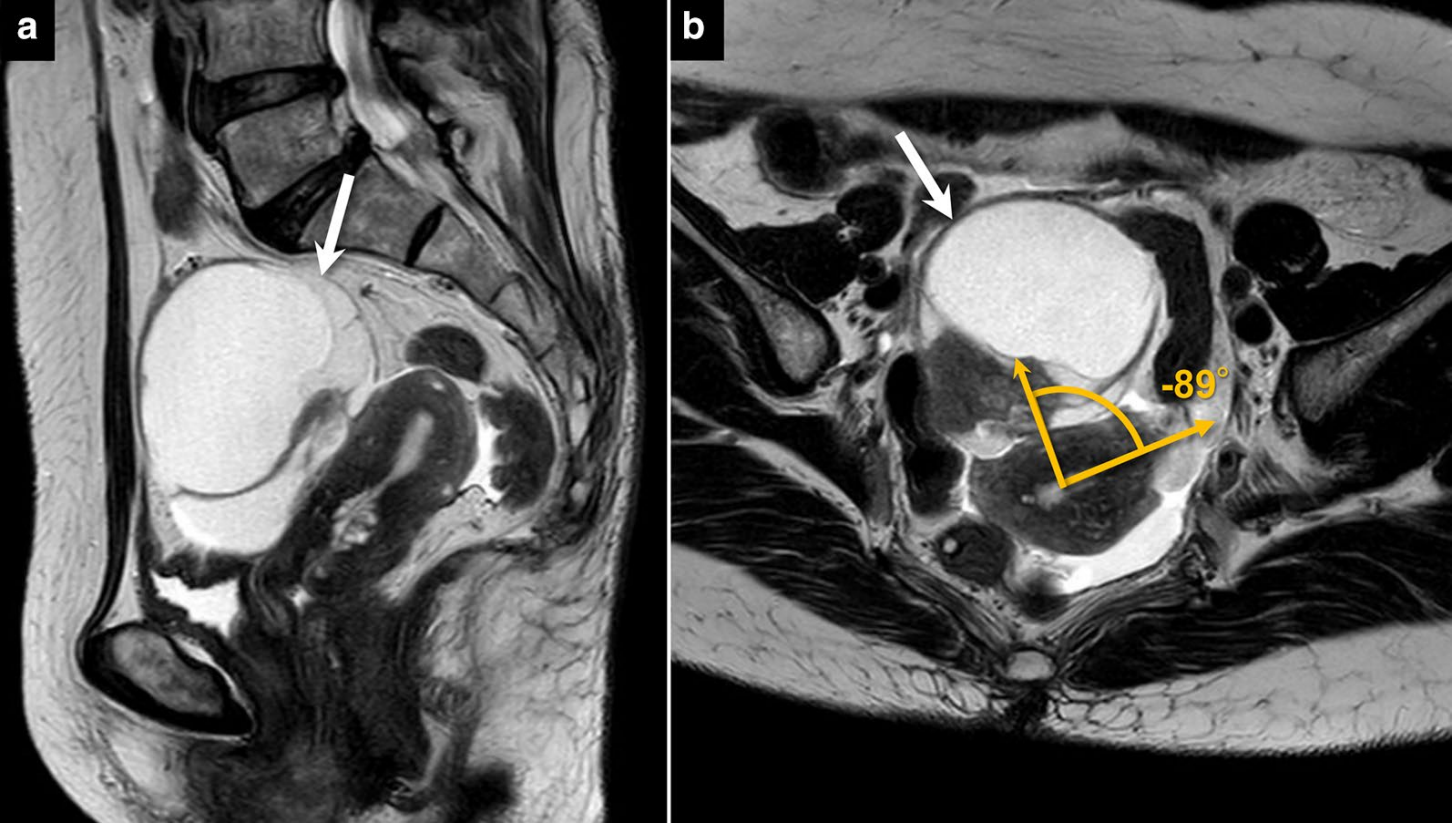
A 78-year-old women with endometrioid carcinoma. **a** Sagittal plane and **b** axial plane to the uterus show a multilocular cystic mass containing solid component (arrows) which was rare but widely adherent to the anterior surface of the uterus without tilt. Pathologically, the mass was derived from the left ovary, and endometriosis was present in the background. The angle with the uterus is measured as –89, as in shown in B

**Fig. 6:**
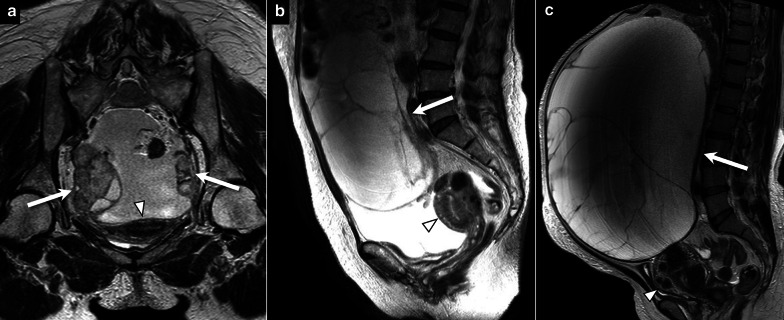
Three cases out of measurement target. **a** A 66-year-old woman with high-grade serous carcinoma, axial plane to the uterus, shows bilateral solid-predominant tumors (arrows), both relatively small, and all interpreters determined that they were not in contact with the uterus (arrowhead). **b** A 73-year-old woman with mucinous carcinoma, sagittal plane to the uterus, shows a multicystic huge tumor (arrow), which was determined by all interpreters not to be in contact with the uterus (arrowhead). **c** A 51-year-old woman with mucinous carcinoma, sagittal plane to the uterus, shows a multicystic huge tumor (arrow) in contact just above the uterus (arrowhead), resulting in discrepancy in ante-post assessment by the interpreters

Figure 4 shows a typical case of CCC of endometriotic cyst origin, in which the tumor was widely adherent to the posterior of the uterus. Figure 5 shows a case of EC derived from an endometriotic cyst, which is rarely located anterior to the uterus. Figure 6 shows cases out of the measurement target: 6A is an HGSC with no attachment to the uterus, 6 B is an MC with no attachment to the uterus, and 6C is an MC case in which the ante-post assessment was inconsistent among interpreters because the tumor was located directly above the uterus.

## Discussion

In this study, we examined the location of several histological types of ovarian cancer in relation to the uterus and revealed that high-grade serous carcinomas (HGSCs) are often not attached to the uterus, and clear cell carcinomas (CCCs) and endometriosis-associated ovarian cancers (EAOCs) are significantly positioned more posteriorly to the uterus than non-endometriosis-associated ovarian cancers (non-EAOCs).

The ovaries typically lie on the peritoneum of the pelvic wall in a shallow fossa, at an angle between the internal and external iliac vessels. The broad ligament is a flat sheet of the peritoneum, extending from the lateral pelvic walls to the uterus and ovaries. The ovarian ligament connects the ovary to the lateral aspect of the uterus, the ovarian suspensory ligament extends from the ovary to the outer abdominal wall, and both are in the broad ligament. Despite these attachments, the ovary is highly mobile and its relationship with the uterus varies. It is assumed that when ovarian tumors occur, their size and presence of endometriosis may affect their position and cause differences in histology; however, there have been no reports examining this phenomenon. Endometriosis is characterized by the presence of endometrial glandular and stromal elements at extrauterine sites, affecting approximately 10% of women of reproductive age [7]. Related neoplasms develop in approximately 1% of patients, mostly occurring in the ovary [8]. It has been suggested that endometriotic cysts provide a microenvironment that enhances neoplastic transformation, including high estrogen levels, causing an increase in cancerous cysts. Iron in the fluid of endometriotic cysts promotes oxidative stress, which can lead to genetic mutations and malignant cysts [8–10]. Several studies have suggested that atypical endometriosis, characterized by atypia, hyperplasia, large nuclei, and an increased nuclear–cytoplasmic ratio, is probably a direct precursor that may result in malignant transformation [11]. Endometriosis is found in 50–74% of CCCs [12–14] and 85–90% of ECs [15–17]. In the present study, as in previous reports, CCC and EC had a high endometriosis complication rate of > 70%. In endometriosis, inflammation occurs in the cul-de-sac, uterosacral ligaments, and ovaries, and chronic cyclical inflammation leads to the formation of adhesions that tether the ovaries to the posterior uterus [18, 19]. On MRI, retropositioned ovaries, which means both ovaries positioned posterior to the cornua of the uterus, appearing adherent to the uterine serosa but not in contact with each other, and kissing ovaries, which means both ovaries in contact in addition to the retropositioned ovaries, are associated with higher intraoperative Revised American Society for Reproductive Medicine classification system endometriosis stages [18]. Although there have been no reports to date showing a correlation between endometriosis severity and the occurrence of EAOCs, considering that untreated endometriosis becomes more severe over time and that EAOCs develop long after the onset of endometriosis, it is understandable that most EAOCs are located at positions similar to the higher stages of endometriosis. For non-EAOCs, MCs were significantly larger than other cancers and occupied the abdominal cavity; thus, they were attached to the uterus even without endometriosis. HGSCs were significantly smaller than other cancers and because they genetically develop de novo from the tubal and/or ovarian surface, they are found to be less frequently attached to the uterus. As the ovarian tumor grows, it moves to the anterior or posterior side of the uterus because there is no space on the side. This is supported by the strong correlation between the absolute values of the measured angles and tumor size in HGSCs, where there was a large variation in size from small to large tumors. As they become even larger, they are expected to protrude above the uterus where there is space. There was no association between previous surgery and the measured angles, and if the uterus was extensively adhered to the anterior abdominal wall during surgery, the tumor moved behind the uterus, but not directly behind the uterus if there was space. Previous research has indicated that women with endometriosis typically have a retroverted uterus [20, 21]. However, this study found that CCCs were significantly more anteverted than HGSCs. Furthermore, this study revealed that women with anteverted uteri had tumors located significantly further posteriorly. These findings suggest that tumor compression and location strongly influence the anterior displacement of the uterine body, as opposed to deformation caused by endometriosis. Additionally, no association was identified between other factors that may be related to uterine position, such as menopausal status and parity, and angles of the tumors.

Our study has several limitations. First, because this study was retrospective, many cases were unmeasurable, resulting in a small number of cases with some histological types. In addition, the data used in this study may have been influenced by uncontrolled biases in the study design (e.g., our hospital being a cancer center, which could attract larger ovarian cancers) or confounding factors (e.g., the presence of uterine myomas or adenomyosis). Furthermore, the evaluation of tumor attachment was influenced by the quality and resolution of the MRI and the expertise of the radiologist interpreting the images, which may have affected accuracy. Second, when the uterus and tumor were in wide attachment, it was difficult to determine the point of contact, resulting in relatively large differences in measured angles. Third, the presence of endometriosis was retrospectively reviewed based on the pathological reports. Therefore, inaccurate, and inconspicuous endometriotic lesions may have been missed, especially in post-chemotherapy cases, which are common in HGSCs. Fourth, this study included only ovarian cancers, among which EAOC is a condition that occurs in a small number of endometriosis patients, and the present results do not apply to ovarian tumors as a whole. Finally, the location of the ovarian tumor alone cannot diagnose a specific histological type, and future evaluation of its diagnostic performance in combination with conventional MRI findings is needed.

In conclusion, high-grade serous carcinomas were significantly smaller than other cancers and are often not attached to the uterus, and endometriosis-associated ovarian cancers, including endometrioid carcinoma and clear cell carcinoma, are located more posteriorly to the uterus than non-endometriosis-associated ovarian cancers.

### Supplementary Information

**Additional file 1**: **Table S1**. Results of the Dunn–Bonferroni post hoc test of the measured angles between ovarian tumor and the uterus according to histological type.

## Data Availability

The datasets used and/or analyzed during the current study are available from the corresponding author on reasonable request.

## Abbreviations

CCC: Clear cell carcinoma
EAOC: Endometriosis-associated ovarian cancer
EC: Endometrioid carcinoma
HGSC: High-grade serous carcinoma
LGSC: Low-grade serous carcinoma
MC: Mucinous carcinoma
SD: Standard deviations
