# Supplementary material for: Differences in the position of endometriosis-associated and non-associated ovarian cancer relative to the uterus
Source: Insights Imaging. 2023 Aug 15;14:136. doi: 10.1186/s13244-023-01468-9 (PMC10425308; doi:10.1186/s13244-023-01468-9)
Supplement: Supplementary file 1 — Additional file 1: Table S1. Results of the Dunn–Bonferroni post hoc test of the measured angles between ovarian tumor and the uterus according to histological type. [file 13244_2023_1468_MOESM1_ESM.pdf]

Differences in the position of endometriosis-associated and non-associated ovarian cancer relative to the uterus

ELECTRONIC SUPPLEMENTARY MATERIAL

Supplemental Table 1.

Results of the Dunn-Bonferroni post hoc test of the measured angles between ovarian tumor and the uterus according to histological type

P value of the Kruskal-Wallis test	*P* value of the Dunn-Bonferroni post hoc test
0.010*	EC vs CCC=0.838, EC vs MC=0.051, EC vs LGSC=0.066, EC vs HGSC=0.077<br>CCC vs MC=0.016*, CCC vs LGSC=0.055, CCC vs HGSC=0.012*<br>MC vs LGSC=0.260, MC vs HGSC=0.525<br>LGSC vs HGSC=0.172

CCC, clear cell carcinoma; EC, endometrioid carcinoma; HGSC, high-grade serous carcinoma; LGSC, low-grade serous carcinoma; MC, mucinous carcinoma; SD, standard deviation; *, $P<0.05$

Insights Imaging (2023) Saida T, Mori K, Ishiguro T, Saida Y, Satoh T, Nakajima T
